# The integration of metabolome and proteome reveals bioactive polyphenols and hispidin in ARTP mutagenized *Phellinus baumii*

**DOI:** 10.1038/s41598-019-52711-7

**Published:** 2019-11-07

**Authors:** Henan Zhang, Ruibing Chen, Jingsong Zhang, Qitao Bu, Wenhan Wang, Yanfang Liu, Qing Li, Ying Guo, Lei Zhang, Yan Yang

**Affiliations:** 1 Institute of Edible Fungi, Shanghai Academy of Agricultural Sciences; National Engineering Research Center of Edible Fungi, Key Laboratory of Edible Fungi Resources and Utilization (South), Ministry of Agriculture, Shanghai, 201403 China; 2 Department of Pharmaceutical Botany, School of Pharmacy, Second Military Medical University, Shanghai, China; 3 Department of Pharmacy, Changzheng Hospital, Second Military Medical University, Shanghai, 200003 China; 4 State Key Laboratory of Subtropical Silviculture, Zhejiang A&F University, Hangzhou, Zhejiang 311300 China

**Keywords:** Metabolic engineering, Applied microbiology

## Abstract

*Phellinus baumii*, also called “Sang Huang” in China, is broadly used as a kind of health food or folk medicine in Asia for its high biological activities, e.g. anti-tumor, anti-oxidation and anti-inflammatory activities. Although some previous studies have indicated that polysaccharides and flavonoids showed the activity of inhibiting tumor cells, the active metabolites of *P. baumii* needs further research. In our study, a stable *P. baumii* mutant (A67), generated by ARTP mutagenesis strategy, showed more significantly inhibiting tumor cells and enhancing antioxidant activity. Our further studies found that the increase of polyphenols content, especially hispidin, was an important reason of the biological activity enhancement of A67. According to the results of the integrated metabolome and proteome study, the increase of polyphenol content was caused by upregulation of the phenylpropanoid biosynthesis. This study expanded the understanding of active compounds and metabolic pathway of *P. baumii*.

## Introduction

*Phellinus baumii*, a well-known fungus in Hymenochaetaceae family, grows on mulberry trees. It is a famous edible mushroom in Asia and it is commonly called “Sang Huang” in China and “Meshimakobu” in Japan^1^. In China, *P. baumii* is even known as “biogold” for its health value and medicinal value^2^. Its fruiting body is traditionally used as a folk medicine for its high biological activities, e.g. anti-tumor cell proliferation^3,4^, anti-inflammatory^5,6^, anti-oxidation^7^, anti-infection^8^. It is even directly used as a food source to strengthen heath and prolong life. Moreover, the extracts have also been found to show hypoglycemic effect^9^.

The medicinal and nutritive value of *P. baumii* have raised more worldwide attention and attracted greater research effort to rediscover this edible fungus. In modern medical research, polyphenols^5,10^, polysaccharides^3,7^ and flavonoids^4,8^ isolated from *P. baumii* are regarded as the major bioactive constituents that show a broad spectrum of health effects and biological activities. Polysaccharides isolated from *P. baumii* have shown that they can inhibit tumor growth and metastasis in previous studies^3^. And *P. baumii* acted as an activator of immune cells, natural killer cells, and macrophages, all of which were shown to remove cancer cells and pathogens^6^. The baicalein, a flavone metabolite, isolated and purified from *P. baumii* showed obvious killing effect on cancer cells^4^. Polyphenols isolated from the ethanolic extract inhibited H1N1, H5N1, and H3N2 neuraminidase activity noncompetitively and reduced the amount of virally-induced cytopathic effect (CPE)^10^. All these studies found polysaccharides and flavonoids have the ability of anti-tumor cell proliferation, and polyphenols have the ability of anti-infection. However, it has not yet been figured out whether polyphenols or flavonoids possess anti-oxidation or anti-tumor cell proliferation bioactivities. Furthermore, it is still unknown what polyphenol or flavonoid monomer contributes to these biological activities. As proteomics and metabolomics are widely used to monitor the processes of biological systems, the integration of proteomics and metabolomics provides a feasible strategy to understand physiologic mechanisms of biodiversity among different samples^11^. By combining proteomics and metabolomics techniques, we can link alterations of protein expression to metabolism, and that will help us to find the reliable clue of bioactive metabolites.

The medicinal value of *P. baumii*, especially its anti-cancer effect, is getting more and more attention, and has triggered over-harvesting of wild *P. baumii* and leaded to scarcity of wild resources^12^. Many studies have shown that the cultivation of fungal mycelia by fermentation has become a major alternative to wild *P. baumii*^13^. As the yield and activity of *P. baumii* obtained by fermentation mainly depend on the quality of the initial *P. baumii* strain^14^, it is very important to find an initial *P. baumii* strain of good quality for fermentation. In previous researches, the SH1 strain was screened out from seven *P. baumii* strains, as it exhibited better growth vitality and higher anti-oxidation activities compared with the other six strains. Because wild strain SH1 is still hard to meet the needs of resources, it is urgent to find other excellent strains to alleviate the resource shortage of *P. baumii* by microbial breeding.

Breeding genetically modified microbes is essential to biotechnological approaches. Mutation are, in nature, the primary derived of evolution when subjected to selection pressure^15^. However, such natural evolution process is slow as mutations occur at relatively low rates^16^. Thus, many different artificial mutagenesis methods to improve the mutation efficiency have been applied, so far by chemical or physical mutagens in industrial and research settings^17^. Recently, a novel strategy, the atmospheric and room temperature plasma (ARTP) mutation approach has been adopted to obtain large population of strains, leading to selective generation of strains of bacteria, fungi, and algae^7^. In 2009, *Streptomyces avermitilis* mutants that produced high levels of avermectins were generated via this approach^18^. When spores were cultivated, >30% of total and ~21% positive mutation rates were evident in the resultant microbes. This led to the generation of a mutant strain that produced >40% more avermectins B1a than WT control strains, with an overall increase in avermectin productivity of 18%^7^. In the past decade, the ARTP mutation system has been widely used and become more stable and efficient.

This study obtained an excellent mutant strain (*P. baumii* A67) that produced significantly higher content of total polyphenols and flavonoids by using ARTP mutagenesis method. The A67 strain also showed better anti-oxidation and anti-cancer cell proliferation activities than SH1 strain, which was expected to substitute wild strains for fermentation. Furtherly, through an integrated analysis, differential proteome-metabolome profiling of A67 and SH1 revealed that activated biosynthesis of phenylpropanoid was an important reason why the polyphenols content and biological activity of A67 were improved.

## Results

### Discovery of a stable *P. baumii* mutant with high yield of polyphenols and flavonoids

**Figure 1:**
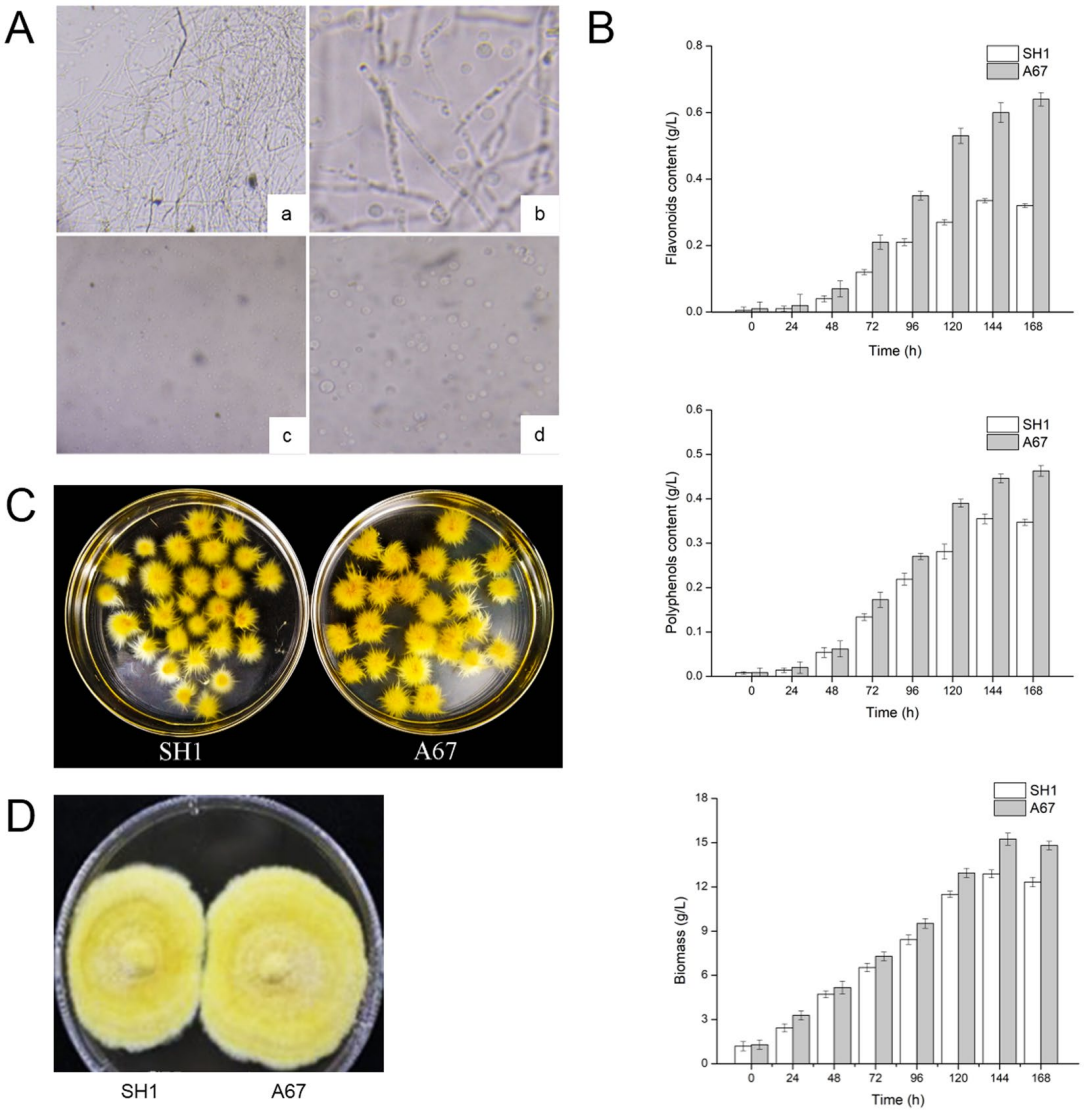
Discovery of the A67 mutant strain with high polyphenols and flavonoids content. (**A**) Preparation of *P. baumii* protoplasts. (a and b), One hour after enzyme treatment; (c and d), three hours after enzyme treatment. (**B**) Comparison of polyphenols, flavonoids, glucose content and biomass between SH1 and A67. (**C**) Comparison of appearance characteristics of SH1 and A67. (**D**) The antagonistic reaction between A67 and SH1.

To generate diverse *P. baumii* mutants efficiently, ARTP mutagenesis strategy was used in this study. The wild strain SH1 was selected as an original *P. baumii* to make protoplast for mutagenesis assay (Fig. 1A). Using this method, a total of 1139 regenerated colonies were obtained, all of which showed various phenotypes in color, growth rate and metabolite content. The colonies with excellent morphological characteristics were isolated for five-generation subculturing. Finally, a total of 4 colonies with stable genetic traits were screened. Through liquid fermentation experiment, only 1 mutant A67 was eligible and promising for further biological activity and mechanism researches, because the mycelia biomass dry weight, the total flavonoids and polyphenols content of A67 were markedly increased relative to the SH1 WT strain. Flavonoids content and polyphenols content of A67 were most significantly increased by 1.87 and 1.33 folds, respectively (Fig. 1B). Compared with SH1, the A67 colonies were yellower and larger (Fig. 1C). Mushroom hyphae can slow the growth of hyphae from other sources during the development stage. This antagonistic reaction allows for assessment of relationship between strains, facilitating mutant strain identification. Herein a clear antagonistic reaction occurred obviously between A67 and SH1, which indicated the genomic differences and the success of mutagenesis (Fig. 1D).

### The inhibitory effects of *P. baumii* ethanol extracts on HepG2 and K562 tumor cells

**Figure 2:**
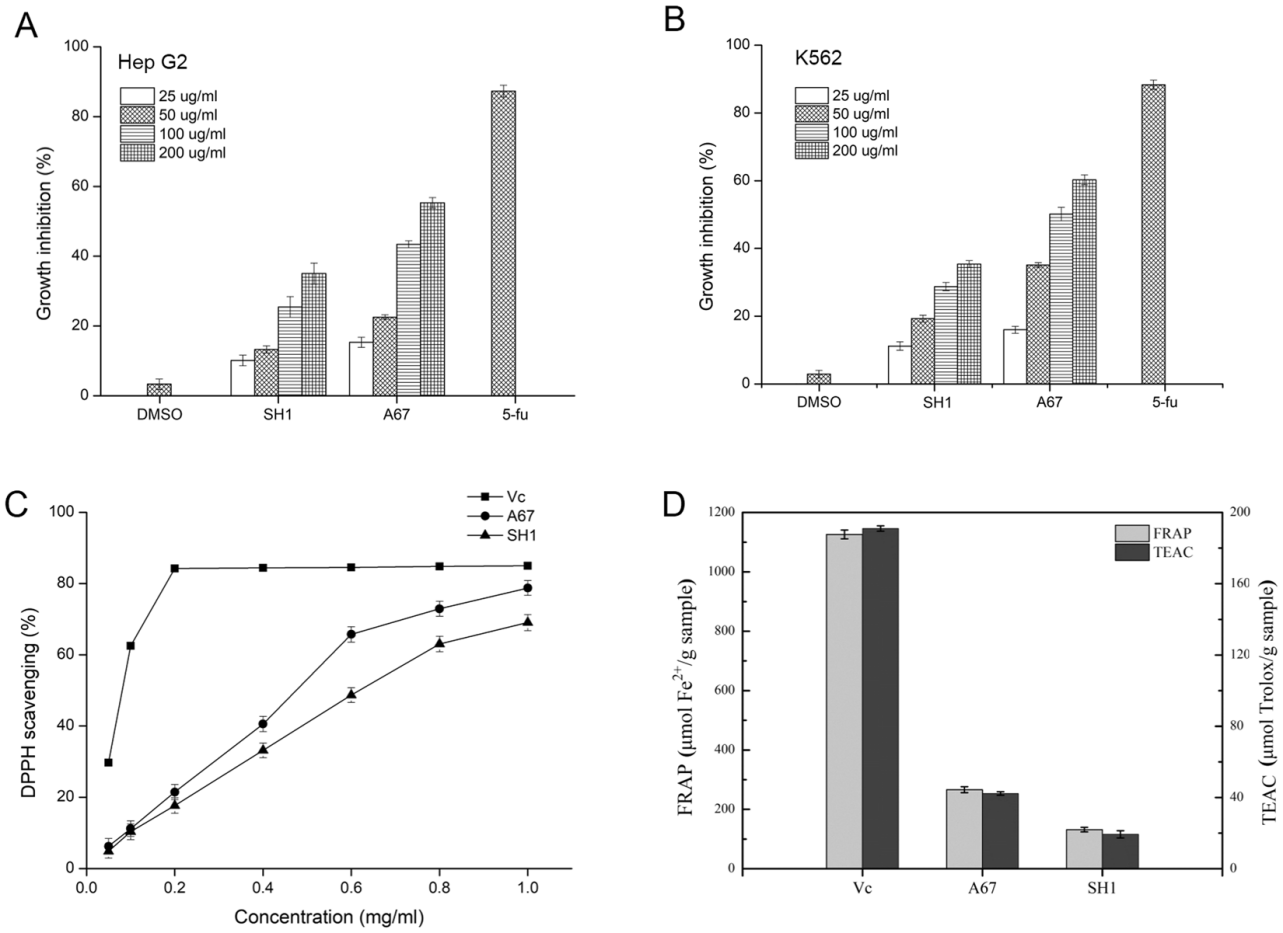
Biological activities of *P. baumii* ethanol extracts at different concentrations *in vitro*. (**A**) Inhibitory effects on tumor cell line HepG2. (**B**) Inhibitory effects on tumor cell line K562. Each value is expressed as means ± SD (n = 3). 5-fluorouracil (5-fu) served as a positive control and DMSO as negative control. (**C**) Comparison of antioxidant effects of *P. baumii* ethanol extracts at different concentrations by DPPH method. (**D**) Comparison of antioxidant effects of *P. baumii* ethanol extracts at different concentrations by FRAP and TEAC method.

HepG2 and K562 cells were used for assessing the ability of *P. baumii* ethanol extracts to inhibit the proliferation of cancer cells. *P. baumii* extracts could inhibited HepG2 and K562 cell proliferation at most tested concentrations (Fig. 2A,B). As shown in Fig. 2A, compared with DMSO, all *P. baumii* extracts (25–200 μg ml^−1^) inhibited the proliferation of HepG2 cell at different concentrations (P < 0.05). At 25 and 200 μg ml^−1^ doses, SH1 extracts showed the lowest and strongest inhibition rate of 10.13% and 35.03% respectively, while A67 extracts suppressed the proliferation of HepG2 cells by 15.34% and 55.32% respectively. Obviously, the ability of A67 extracts to inhibit HepG2 cells proliferation was significantly stronger than that of SH1 extracts (P < 0.05). At 200 μg ml^−1^, the ability of A67 extracts inhibiting tumor cell proliferation was 1.58 folds higher than that of SH1 extracts. As shown in Fig. 2B, K562 cells were also incubated with extracts of 25, 50, 100 and 200 μg ml^−1^ and the extracts mediated the dose-dependent inhibition of K562 proliferation. Results indicated that the strongest inhibition rate of 60.27% was at the highest A67 extracts concentration of 200 μg ml^−1^, which were 1.70 folds higher than that of SH1. The IC_50_ concentrations of *P. baumii* ethanol extracts in two tumor cell lines were determined (Table 1). All the results indicated that *P. baumii* extracts of A67 strain showed stronger suppression of tumor cell activity relative to WT SH1 strain.

**Table 1:** IC50 concentration of *P. baumii* ethanol extracts in tumor cell lines.

Strain	IC_50_ (μg ml^−1^)	
	HepG2	K562
SH1	263.75	164.04
A67	202.57	99.65

### Antioxidant effects of *P. baumii* ethanol extracts *in vitro*

The DPPH, FRAP and TEAC assays were used to analyzed *P. baumii* extract antioxidant activity. DPPH radicals is commonly used to assess free radical scavenging activity of antioxidants, with hydrogen molecule donation activity being attributable to DPPH scavenging in this assay. We observed a dose-dependent increase in free radical scavenging of DPPH by SH1 and A67 extracts (Fig. 2C). At the 1.0 mg ml^−1^ concentration, these two extract types had 69.06% and 78.76%, DPPH scavenging activity, respectively, with A67 extracts having higher scavenging activity than that SH1 extracts (P < 0.05). The FRAP assay relies upon antioxidant-mediated reduction Fe^+3^ to Fe^2+^ when 2,4,6-tris-(2-pridyl)-s-triazine (TPTZ) is present, resulting in the formation of a blue-colored compound visible via assessing absorbance at 593 nm. In this assay, reductions in absorbance are directly correlated with antioxidant ability of extracts. Similar to the results obtained from the DPPH, the A67 extracts showed relatively strong ferric ion-reducing activity with FRAP value of 266.2 μmol Fe^2+^ g^−1^, while SH1 extracts showed lower activity with FRAP value of 131.82 μmol Fe^2+^ g^−1^ (Fig. 2D). Also, A67 extracts exhibited more significant antioxidant activity with TEAC value of 42.28 μmol Trolox g^−1^ than that of SH1 extracts (Fig. 2D). All the results indicated that *P. baumii* extracts of A67 strain had a stronger antioxidant activity compared with SH1 strain in this study.

### Differences of metabolome between SH1 and A67 strains

**Figure 3:**
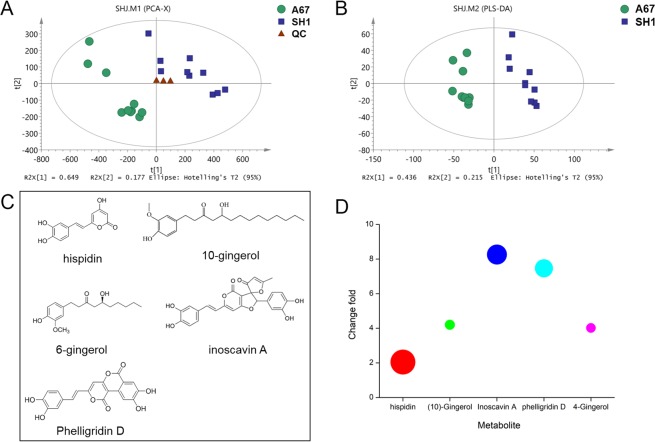
Differences of metabolite accumulation between SH1 and A67 strains. (**A**) PCA scores plots of nine SH1 and nine A67 strains based on the extract spectral data of UPLC-LTQ Orbitrap and announced differential metabolites. (**B**) PLS-DA scores plots of nine SH1 and nine A67 strains based on the extract spectral data of UPLC-LTQ Orbitrap and announced differential metabolites. (**C**)The structure of 5 differential polyphenol metabolites. (**D**) The changes and relative content of five differential metabolites. Different colors represent different metabolites, the size of the circle represents the relative content and the Y-axis represents the fold changes.

To figure out the reason why A67 showed stronger antioxidant and proliferation inhibitory activity, a metabolome analysis of differential metabolites was adopted in this study. The QC were utilized to determine the best detection conditions, with Supplementary Fig. S1 displaying sample base peak intensity (BPI) chromatograms as generated using optimized ESI+ and ESI− conditions. It is easy to find from BPI chromatograms that some peaks were obviously higher in the A67 than SH1, which is highlighted in Supplementary Fig. S1. After data normalization, a total of 3695 and 1872 peaks were obtained from spectral data of ESI+ and ESI−. The PCA and PLS-DA scores plots constructed with the resulting peaks indicated a clear separation between the SH1 group and A67 group (Fig. 3A,B), and parameters of PCA and PLS-DA models were listed in Supplementary Table S1. Based on the PLS-DA model and appropriate statistical testing, a total of 96 differential metabolites were selected with a *p* value less than 0.01 and a variable influence on projection (VIP) value more than 1.5 between the SH1 and A67, and were annotated with the available reference standards as well as databases (Supplementary Table S2). Of these differential metabolites, the content of 62 metabolites was increased, while the content of 18 metabolites was markedly reduced (P < 0.01). Interestingly, all 5 identified polyphenol metabolites (hispidin, 10-gingerol, inoscavin A, phelligridin D, and 4-gingerol) were accumulated much higher (Fig. 3C). Although the content of hispidin was only increased by 2.05 folds, the relative content was much more abundant than the other four polyphenol metabolites (Fig. 3D). To validate the structure of hispidin, the compound was extracted from A67, followed by semi-preparative HPLC for high-resolution 1D and 2D NMR analysis. The purified product was confirmed to be hispidin based on these NMR spectra and literature reference (Supplementary Fig. S2)^19^. These results implied that hispidin might be the crucial active polyphenol metabolite that exerted antioxidant and anti-tumor cell proliferation activities.

### The phenolic metabolite hispidin plays an important role in suppression activity on tumor cells proliferation and antioxidant activity

**Figure 4:**
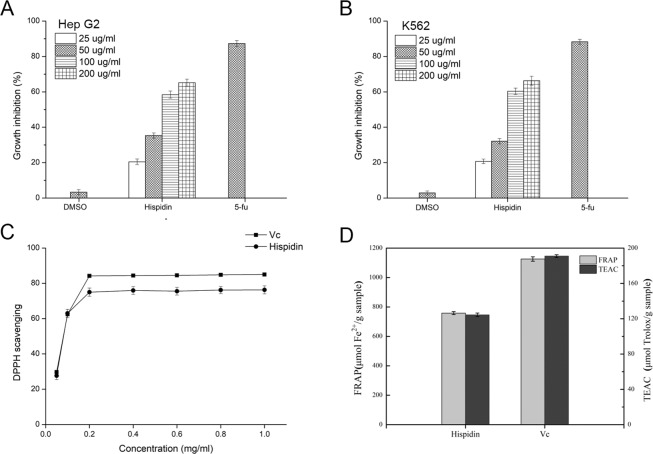
Biological activities of hispidin. Inhibitory effects of hispidin at different concentrations on (**A**) HepG2 and (**B**) K562 cell lines *in vitro*. Antioxidant effects of hispidin at different concentrations by (**C**) DPPH method and (**D**) FRAP and TEAC method.

In order to verify the activity of hispidin, an activity verification experiment for inhibiting tumor cell proliferation and antioxidation was performed. HepG2 and K562 cells were used to analyzed how hispidin impact cancer cell proliferation. Hispidin obviously could inhibited HepG2 and K562 cell proliferation in a dose-dependent manner. Similar to the 5-fluorouracil (5-Fu), hispidin exhibited very strong inhibitory effect. Hispidin demonstrated the inhibition rate of 65.21% and 66.31% on HepG2 (Fig. 4A) and K562 (Fig. 4B) at 100 µg mL^−1^, respectively. The above results showed hispidin’s suppression on tumor cells. The DPPH, FRAP and TEAC approaches were used to assess hispidin antioxidant activity. As shown in Fig. 4C, DPPH radical scavenging by hispidin rose in a dose-dependent fashion. At 0.2 mg ml^−1^, hispidin and Vc (positive control) DPPH scavenging levels highly reached 75.01% and 84.22%, respectively. Similar to the results obtained from the DPPH, hispidin showed extremely strong ferric ion-reducing activity with FRAP value of 758.29 μmol Fe^2+^ g^−1^ and antioxidant activities with a 124.46 μmol Trolox g^−1^ TEAC value, which were 67.34% and 65.16% times than the antioxidant capacity of Vc, respectively (Fig. 4D). These results suggested that hispidin, like Vc, significantly reduced oxidative stress *in vitro*.

### Integration of the metabolome and proteome demonstrates improved polyphenolic metabolism in A67 strain

**Figure 5:**
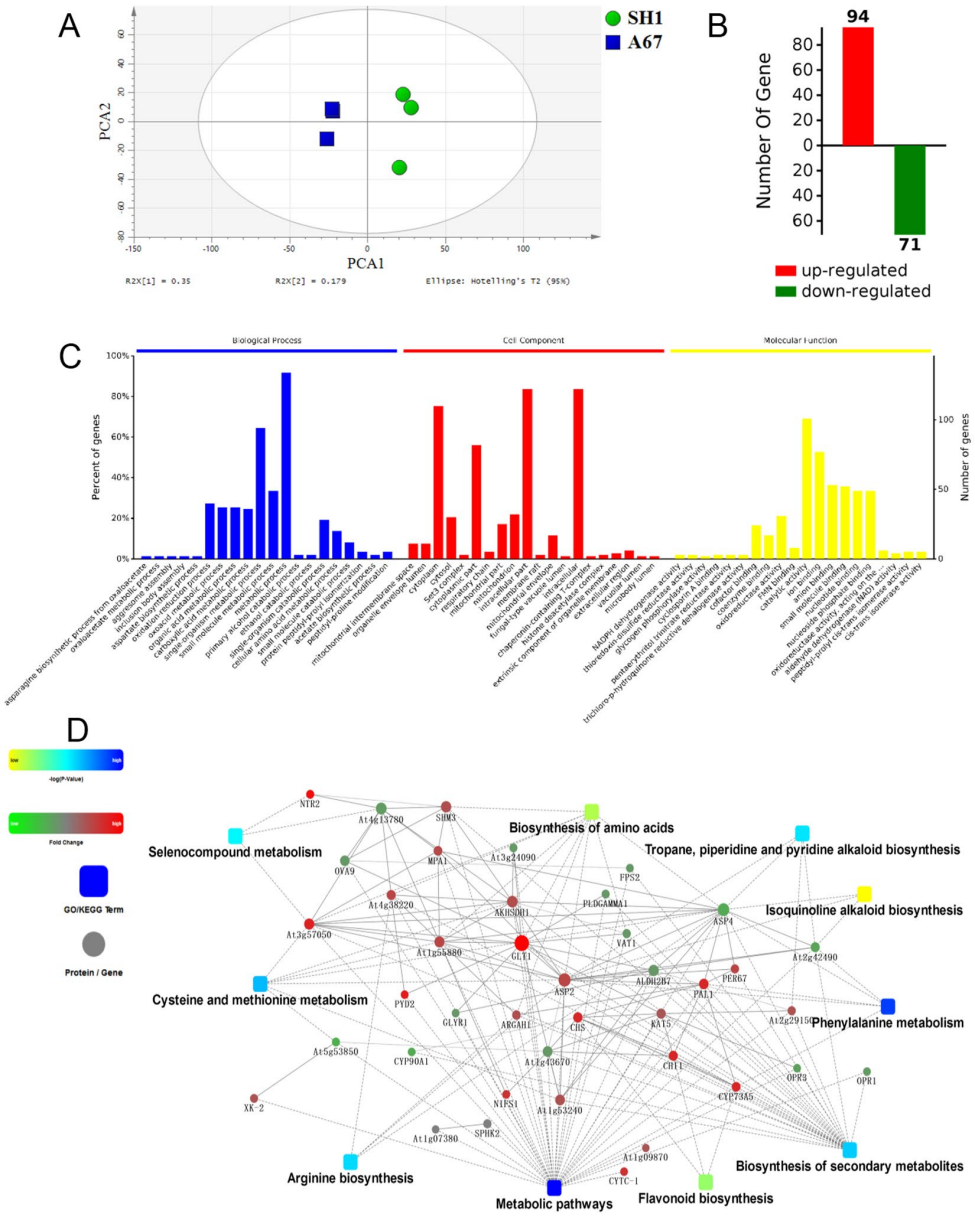
Differences of proteome between SH1 and A67 strains. (**A**) PLS-DA scores plots. (**B**) Number of differentially expressed proteins. (**C**) GO analysis of differential proteins. (**D**) Correlation network between differential metabolites and proteins.

To explore secondary metabolism reprogramming in A67 strain, the differential metabolites provided distinct pools of candidate metabolites involved in antioxidant activity. The essential cause of the enhanced antioxidant and tumor cell growth inhibitory activity of A67, however, had yet to be identified. To facilitate further mechanistic analyses of this activity, an integrated network of differential metabolites and proteins was constructed. Firstly, LC-MS/MS combined with SCX was used to detect proteins differentially expressed in A67 strains. After analysis of iTRAQ labeled and fractionated fractions by LC-MS/MS and annotation by the UniProt Knowledgebase, a total of 2195 peptides matching 436 proteins (≥1 peptide) were found through sample analysis. PCA was employed to display variation between SH1 and A67 strains. Plots representing overall experimental samples and distinguishing different groups through different colors exhibited clear sample separation (Fig. 5A). Plots corresponding to A67 strain samples were well-clustered and distinct from those of the SH1 strain. The results indicated the good reproducibility of the experiment samples and the obvious variation between two groups. With a 1.2-fold change threshold and a P value <0.05 as a cutoff, a total of 165 proteins were extracted, of which 94 and 71 were up- and down-regulated, respectively (Supplementary Table S3 and Fig. 5B). The ratios of the iTRAQ reporter ions between the SH1 and A67 samples were used to establish fold change values (Supplementary Table S3). At the same time, all the differences in protein Gene Ontology (GO) enrichment and significant number were summarized (P < 0.05). In Fig. 5C, these differentially expressed proteins were classified based upon biological process (BP), cell component (CC) and molecular function (MF), but the most differential proteins were clustered into the BP category. As shown in Fig. 5C, seven subgroups in BP group, “metabolic process”, “single-organism metabolic process”, “small molecule metabolic process”, “oxidation-reduction process”, “oxoacid metabolic process”, “organic acid metabolic process”, and “carboxylic acid metabolic process” obviously possessed more members than others, consistent with enhanced metabolic activities. Moreover, in all specific metabolic classifications, the “oxidation-reduction process” contained the most differential proteins. These results suggested that the most of differentially expressed proteins were associated with “oxidation-reduction process” in A67 was responsible for the increase in its antioxidant activity. Using OmicsBean, we constructed a correlation network based on 96 significantly different metabolites and 165 significantly different proteins. This interaction network showed top ten metabolic pathways of KEGG with significant differences and the interacting proteins (annotated name in the *Arabidopsis thaliana*). As shown in Fig. 5D, the visualized network consisted of three elements: the squares representing metabolic pathways, the circles representing proteins, and the dotted lines representing interactions. The square represents the metabolism of phenylpropanoid (dark blue), indicating the most significant difference occurred in the phenylpropanoid metabolic pathway, which was correlated with three key up-regulated enzymes in the phenylpropanoid metabolic pathway, i.e. PAL, CHI and CYP73A (C4H). It was proposed that hispidin and many other polyphenols are derived from two different pathway. Hispidin was derived from phenylpropanoid and biosynthesized by the condensation of one molecule of caffeoyl-CoA and two molecules of acetyl-CoA^20^. A 4-hydroxy-6-methyl-2-pyrone-mediated pathway of biosynthesis has been proposed, with three acetyl-CoA and one 3,4-dihydroxybenzoyl-CoA (3,4-dihydroxy benzaldehyde)^21^. For phenylpropanoid pathway, it was also well known that phenylalanine is gradually transformed into caffeoyl-CoA by PAL, C4H, 4CL and C3H^22,23^. Thus, in the A67 strains, the up-regulation of phenylpropanoid pathway might lead to an increase in the accumulation of polyphenolic metabolites (including hispidin), the biological activities of A67, therefore, was enhanced.

## Discussion

**Figure 6:**
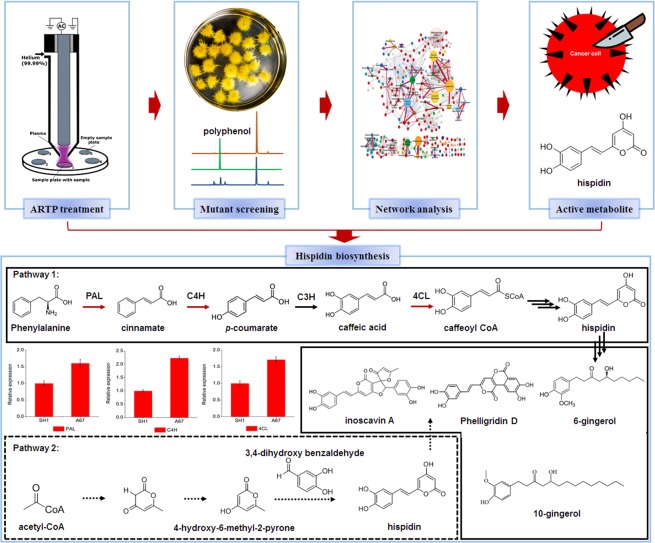
The research strategy that integrates omics data to explore bioactive products and biosynthesis pathway analysis of hispidin. The solid lines represent the confirmed metabolic pathway. Dotted lines represent presumed metabolic pathway. The red arrows represent the upregulated steps in A67 strain. Dotted arrows represent the presumed steps. The histograms represent the relative expression level of the proteins in the SH1 and A67 strains.

In this study, we obtained the high-quality strain A67 by using the ARTP mutagenesis strategy, which showed better inhibition of tumor cell proliferation and enhancement of antioxidant activity. A correlation network constructed by the integration of metabolomes and proteomes showed that the polyphenols metabolism represented by hispidin was up-regulated (Fig. 6). Furtherly, purified and structurally defined hispidin showed significant inhibition of tumor cell proliferation and enhancement of antioxidant activity. Therefore, we believe that in the mutant strain A67 of *P. baumii*, ARTP treatment activates the metabolism of polyphenols and accumulates more active metabolites, such as hispidin, making A67 show better activities.

To understand why the polyphenols content was significantly increased in A67, the protein expression of the hispidin metabolic pathway was analyzed. Compared with SH1, PAL, C4H and 4CL expression were markedly upregulated in the A67 strain, and they were all upstream steps of the phenylpropanoid. Two different biosynthetic pathways of hispidin have been proposed in previous research (Fig. 6). Pathway 1: Hispidin is derived from phenylpropanoid. Tracer studies in *Polyporus schweinizii* showed incorporation of D, L-Phe, cinnamate, *p*-coumarate, and caffeate into the styryl unit, with malonate and acetate being efficiently incorporated into the hispidin pyrone ring^20,24^. Pathway 2: 4-hydroxy-6-methyl-2-pyrone-mediated biosynthetic pathway, with three 3,4-dihydroxybenzoyl-CoA molecule being involved in the formation thereof, has been proposed^21^. In this study, up-regulation of the phenylpropanoid pathway leaded to an increase in the content of the hispidin precursor compound, caproyl-CoA. Finally, the polyphenols content was significantly increased because of the highly accumulated hispidin. Taken together, these results verified that biosynthetic pathway 1 of hispidin was present in *P. baumii* and was up-regulated in mutant A67.

## Material and Methods

### Strains

The wild type (SH1) and mutant *P. baumii* strains were obtained from the Preservation Center of Fungi, Institute of Edible Fungi, Shanghai Academy of Agricultural Sciences.

The *P. baumii* A67 mutant strain (CGMCC No.12242) was obtained by ARTP mutagenesis and preserved in China General Microbiological Culture Collection Center, CGMCC (Beijing, China). Positive colonies should exhibit faster mycelia growth rate compared with the CK’s (SH1). After five generations, the positive colonies with genetic stability of the mycelium growth were selected. Random Amplified Polymorphic DNA (RAPD) Analysis was used to screen and identify mutants from a molecular genetic perspective^25^. After a series of screening and identification, a total of 4 positive mutants with stable genetic traits were screened. Through liquid fermentation experiment, positive mutant A67 was eligible and promising for further biological activity and mechanism researches, because of the mycelia biomass dry weight, the total flavonoids and polyphenols content of A67 were significantly higher than those of the wild strain SH1.

### Media and cultural conditions

*P. baumii* strains were incubated on PDA (Potato Dextrose Agar, BD, USA) at 26 °C. Five ~5 mm × 5 mm mycelia sections were used for seed cultures inoculation in 250 mL flasks containing 100 mL of 24 g/L PDB basal medium (BD, USA) and 10 g/L mulberry shoot powder, and shaked (130 rpm) for 7 days at 26 °C. Fermentation culture was conducted in a 1000 mL flask that held 400 mL fermentation medium [glucose (20 g/L, yeast extract (10 g/L), KH_2_PO_4_ (1 g/L), MgSO_4_.7H_2_O (1 g/L), mulberry shoot powder (10 g/L)] to which a 10% seed culture volume was added. Fermentation the proceeded for 7 d at 26 °C.

### Preparation of protoplasts of *P. baumii*

The preparation method of *P. baumii* protoplasts from the liquid fermentation mycelia was based on our published article^26^. Lysozyme and driselase were used to prepare protoplasts under 30 °C. The respective concentrations of lysozyme and driselase were 1% (w/v) and 0.25% (w/v). The optimum concentration of *P. baumii* was 6.9 × 10^6^ per milliliter with the 3 h incubation time.

### Determination of biomass, intracellular polyphenols, total flavonoids and glucose

The dry weight of mycelium was determined by freeze drying to get a constant dry biomass weight. The total polyphenols content was determined by Folin-Ciocalteu method using microtiter 96-well plate at 700 nm^27^. Total flavonoids production was assayed using UV colorimetric method according to the published literature^11^. The method described by Saqib &Whitney was used to determine the content of glucose in this study^28^.

### The preparation of the extracts of *P. baumii*

Freeze-dried and ground mycelia powder (1 g) was soaked and extracted using 10 × volume of 80% ethanol and processed by ultrasound for 2 h. Samples were then spun for 15 min at 10,000 × g collect extracts, which were concentrated by a rotary evaporator, stored at −80 °C.

### Tumor cell lines and culture

K562 and HepG2 cells were grown in RPMI-1640 medium containing 10% FBS and 100 mg mL^−1^ streptomycin/ 100 U L^−1^ penicillin. Breast cancer cells MCF-7 were cultured in MEM Eagle media containing 10% FBS as well as penicillin/streptomycin and 0.01 mg mL^−1^ bovine insulin. Cells were incubated at 37 °C with 5% CO_2_.

### Inhibitory effects of extracts and hispidin from *P. baumii* on tumor cell proliferation

Once 80% confluency, tumor cells were collected, resuspended at 1 × 10^4^ mL^−1^, a dded to 96 well plates in a 199 volume, and treated with 1 uL volumes of appropriate extracts or hispidin (isolated and identified in our laboratory) were dissolved inusing DMSO to the final concentrations of at 25, 50, 100 and 200 µg mL-1. HepG2 tumor cells (1 × 104 cells mL-1, 199 µL) and test sample (1 µL) were added to each well of a 96-well plate. 5-Fu (50 µg mL) and 0.5% DMSO served as As positive and negative controls, cells were instead treated with 5-Fu (50 µg mL) and 0.5% DMSO, respectively. After 72 h, cell media was exchanged for phenol red-free media (180 uL) to which 20 µL alamar Blue was added prior to an additional 1 h incubation. A plate reader was then used for assessing absorbance at 570 nm and 600 nm. As K562 cells were not adherent, they were first spun down for 6 min at 720 rpm before being resuspended in the phenol red-free/alamar Blue media and being shaken at 80 rpm for 10 min. Samples were assessed in triplicate, with data as means ± SD. Inhibition rate was determined to be equal to [1 − (117216 × A_570(sample)_ − 80856 × A_600(sample)_)/(117216 × A_570(control)_ _−_ 80856 × A_600(control)_)] × 100%^29^.

### DPPH radical scavenging ability

Sample DPPH free radical scavenging was analyzed with a 6 × 105 M DPPH solution prepared in methanol. A total of 100 uL of appropriate samples was added to a 2900 uL volume of this solution, and samples were incubated for 1 h in the dark after which absorbance at 517 nm was analyzed. Higher absorbance values coincided with poorer free radical scavenging activity. For control samples, 80% methanol was added in lieu of a sample. The following formula was used for analysis: DPPH radical scavenging activity = [1 − (S − S_B_)/(C − C_B_)] × 100%, where S, S_B_, C, and C_B_ were the absorbances of the sample, the blank sample, the control, and the blank control, respectively^30^.

### Ferric reducing antioxidant power assay

A total of 200 uL of an appropriate sample was mixed with 200 uL 5 mM FeSO_4_, after which 200 uL 1% (v/v) H_2_O_2_ was added into while samples were steadily mixed for 1 h at room temperature. Absorbance at 510 nm was then assessed, with the following formula used for analysis: scavenging activity = (1 − A/A0) × 100%, with A and A_0_ corresponding to sample and control absorbance readings, respectively. As a positive control, vitamin C (Vc) was used^30^.

### Trolox equivalent antioxidant capacity assay

Extract and hispidin TEAC was compared using the ABST^+^ radical generated via ABTS oxidation with potassium persulphate. Trodox was used to generate a calibration curve with which sample TEAC values could be determined. The procedures for measuring TEAC values were carried out in accordance to previous method^31^.

### LC/MS analysis and statistical analysis

Refering to previous research method^32^, the ACQUITY UHPLC system Ultimate 3000 (Thermo Fisher Scientific, Waltham, MA, USA) coupled with LTQ Orbitrap MS (Thermo Fisher Scientific, Waltham, MA, USA) was used for metabolic profiling in both ESI^+^ and ESI^−^ ion modes. Metabolite separation in the positive ion mode was performed with a 2.1 × 100 mm ACQUITYTM 1.7 μm BEH C8 column, using 0.1% formic acid (A) and acetonitrile (B) as mobile phases. Elution was conducted with the following linear gradient: 1 min of 5% B, and a linear increase to 100% over 24 min, holding at this concentration for 4 min. Next, A decrease from 100% to 5% B was conducted from 28 to 28.1 min, with holding at 5% B from 28.1 to 30 min. The total sample analysis time was 30 min. For the negative ion mode, a 2.1 × 100 mm ACQUITYTM 1.8 μm HSS T3 column was used for metabolite separation. In this analysis, the mobile phase was composed of 6.5 mM ammonium bicarbonate in water (C) and 6.5 mM ammonium bicarbonate in 95% methanol (D). For this approach, samples were analyzed over 25 min with linear elution gradients as follows:5% D for 1 min, then a linear increase to 100% D from 1 to 18 min followed by holding at 100% D for 4 min. Next, the concentration was decreased from 100% to 5% D from 22 to 22.1 min, followed by holding at 5% D for 22.1 to 25 min. Columns were warmed to 50 °C with a flow rate of 0.35 ml min^−1^. A 5 uL injection volume was used, with MS settings as follows: 350 °C and 360 °C capillary temperature and 3.5 kV and 3.0 kV spray voltage for positive and negative ion modes, respectively. A 50 to 1000 m/z mass scan range was used.

The XCMS software was used for MS data analysis, yielding information regarding mass, chromatographs, and retention time. Data were normalized to total peak areas for a given sample with Excel 2007 followed by import into SIMCA (v14.0, Umetrics, Umea, Sweden), which was used to conduct principal component analysis (PCA) and partial least-squares discriminant analysis (PLS-DA). SIMCA was also used to mean-center and unit variance (UV)-scale data. The 95% CI of modeled variation was defined based on the Hotelling’s T2 region, which presents as an ellipse on plots. R2X or R2Y and Q2 values define model quality, with R2X or R2Y corresponding to the amount of data explained by models. Q2 is a predictability indicator calculated based upon cross-validation procedures, corresponding to the amount of data variance predicted by the model. SIMCA was used for seven-round cross-validation in order to determine the best number of principal components so as to reduce the risk of overfitting.

### Identification of differential metabolites

The VIP significant threshold identified using the PLS-DA model was used for differential metabolite identification, with metabolites that had a VIP value >1.5 and a p value <0.01 in a two-tailed Student’s t-test being included as differential metabolites.

We identified differential metabolites using the One-step Solution for Identification of Small Molecules in Metabolomics Studies software that was designed by the Dalian Institute of Chemical Physics, Chinese Academy of Sciences and Dalian ChemData Solution Information Technology Co., Ltd to identify differential metabolites. The HMDB and METLIN databases of reference materials that was also designed by these organizations were used^32^.

### Protein extraction

Proteins were extracted as described by Isaacson^33^ with some modification. Mycelia were ground to a fine powder, of which 1 g was added to 10 mL cold acetone containing 10% TCA at −20 °C for 1 h. Samples were then spun at 15,000 × g for 15 min at 4 °C, samples were resuspended using cold acetone for 1 h at −20 °C, and this step was repeated. After spinning down again, samples were freeze-dried in a vacuum before suspension with cold phenol extraction buffer, and addition of an equivalent volume of Tris-HCl (pH = 7.5) saturated with phenol, and the mixture was shaken for 30 min at 4 °C. We collected the upper phenolic phase after spinning for 30 min at 5000 × g at 4 °C and added equal phenol extraction buffer to the collected phenolic phase and collected the upper phenolic phase again, repeated 3 times. Then we added 5 times the volume of cold 0.1 M ammonium acetate in methanol to the collected phenol phase and stored at −20 °C for 1 h. We added 2 times the volume (of the most recent phenolic phase) of ice-cold methanol for pellet washing that was collected through a centrifugation at 5,000 × g for 30 min at 4 °C. It’s necessary to wash the pellet twice with methanol and acetone. Dissolved, washed and dried deposit was then kept in lysis solution at 30 °C for 1 h. The supernatant after centrifuging twice at 15,000 × g for 15 min at room temperature was collected. The supernatant was the extracted protein solution. A BCA approach was used to quantify protein contents prior to storage at −80 °C for iTRAQ assessment.

### Protein sample preparation and labeling

Added five times the volume of cold acetone to 100 μg protein, collected and dried the deposit after centrifugation at 12,000 rpm for 15 min at 4 °C. The pellet was resuspended by 50 μL dissolution buffer and 4 μL reducing reagent at 60 °C for 1 h. Added 2 μL cysteine-blocking reagent at room temperature for 10 min and purified the protein solution by a 10 kDa ultrafiltration tube. Using 100 μL dissolution buffer to wash protein three times. Added 50 μL sequencing-grade trypsin (50 ng μL^−1^) and incubated at 37 °C for 12 h. Then the sample was centrifuged by 12,000 rpm for 20 min, and the peptide was collected. Transferred the filter units to a new collection tube and added 50 μL dissolution buffers to centrifuge the tube again. Combined the two filtered solution.

Added 150 μL of ethanol to each centrifuged iTRAQ reagent vial under room-temperature. Combined prepared iTRAQ reagent with 50 μL of peptide solution (100 μg) and incubated at room temperature for 2 h. Added 100 μL water and vortexed to stop the labeling reaction. Spun and collected the solution, and then dried the sample in a vacuum freeze dryer for iTRAQ analysis.

### 2D-LC-MS/MS analysis

Resuspended the labeled peptide with 100 μL buffer A (pH = 10, 0.1% ammonium formate and 2% acetonitrile in water) to prepare for analysis. The RPLC was employed on the Agilent 1200 HPLC System. We used 215 nm and 280 nm UV detection with an analytical guard column (4.6 × 12.5 mm 5 μm) and a separation column (Narrow-Bore 2.1 × 150 mm 5 μm). A flow rate of 0.3 mL min^−1^ was used to separate samples with a nonlinear binary gradient transitioning grom buffer A to B (pH = 10, 20 mM ammonium formate in 80% acetonitrile) as follows. 2% B from 0 to 3 min, 2 ~ 6% B for 0.01 min, 6 ~ 25% B for 40 min, 25 ~ 38% B for 10 min, 38 ~ 90% B for 0.01 min, 90% B for 10 min, 90 ~ 2% B for 0.01 min and 2% B for 5 min. Collected fractions every 4.5 min from 4.5 to 45 min and only one fraction from 46 to 50 min. In total 10 segments were dried in a vacuum freeze dryer for LC-MS/MS analysis.

### RPLC-MSMS analysis

Refer to previous research method^34^, Nano-RPLC buffer A (0.1% formic acid and 2% acetonitrile in water) was used to resuspend samples to prepare for analysis. Online Nano-RPLC with an Eksigent nanoLC-Ultra™ 2D System (AB SCIEX) was then conducted, with samples being added into the C18 nanoLC trap column (100 µm × 3 cm, 3 µm, 150 Å) followed by washing using Nano-RPLC Buffer A (10 min, 2 μL min^−1^). Next, over 70 min an elution gradient of 5 ~ 35% acetonitrile (0.1% formic acid) was applied on the analytical ChromXP C18 column (75 μm x 15 cm, 3 μm 120 Å) using a spray tip. A Triple TOF 5600 System (AB SCIEX, USA) fitted with a Nanospray III source (AB SCIEX, USA) and a pulled quartz tip emitter (New Objectives, USA) was used for data acquisition using 2.5 kV ion spray voltage, 30 PSI curtain gas, 5 PSI, nebulizer gas and a 150 °C interface heater temperature. During information dependent acquisition (IDA), 250 ms survey scans were conducted for MS data with up to 35 product ion scans being obtained for MS^2^ data per IDA circle when exceeding a 150 counts per second (counts s^−1^) threshold with a 2+ to 5+ charge-state. A total fixed cycle time of 2.5 s was used, with the application of a rolling collision energy setting to all precursor ions for collision-induced dissociation (CID). Dynamic exclusion was set for ½ of peak width (18 s).

### Protein identification, quantification and bioinformatic analysis

Data was processed with Protein Pilot Software v. 5.0 (AB SCIEX, USA) against Agaricomycetes database by the Paragon algorithm^35^. Tandem MS data were used to identify proteins through comparisons with theoretical data. Differentially expressed proteins, were identified based upon a combination of LC-MS/MS combined with Strong Cation Exchange (SCX). As mentioned before, equal amounts of proteins from SH1 and that from matched A67 were subjected to trypsin-mediated digestion prior to mixing these peptides for labeling using iTRAQ reagents (119 as an internal control; 113, 114, and 115 for the SH1 strains; 116, 117, and 118 for the A67 strains). The ration of iTRAQ reporter ions between SH1 and A67 samples was used to assess protein fold change values, with 1.2-fold change and *p* < 0.05 as the criteria for differential protein identification. The GO enrichment analysis in this study used David 6.7 (http://david.abcc.ncifcrf.gov/) and Quick GO (http://www.ebi.ac.uk/QuickGO/) to examine GO annotations for individual proteins and to screen for particular phenotypes.

## Supplementary information

Supplementary information

Dataset Table S2

Dataset Table S3

## Data Availability

The datasets generated during and/or analyzed during the current study are available from the corresponding authors on reasonable request.
