# Supplementary material for: The integration of metabolome and proteome reveals bioactive polyphenols and hispidin in ARTP mutagenized Phellinus baumii
Source: Sci Rep. 2019 Nov 7;9:16172. doi: 10.1038/s41598-019-52711-7 (PMC6838117; doi:10.1038/s41598-019-52711-7)
Supplement: Supplementary file 1 — Supplementary information [file 41598_2019_52711_MOESM1_ESM.pdf]

The integration of metabolome and proteome reveals bioactive polyphenols and hispidin in ARTP mutagenized *Phellinus baumii*

Henan Zhang[1,#], **Ruibing Chen**[2,#], **Jingsong Zhang**[1], **Qitao Bu**[2], **Wenhan Wang**[1], **Yanfang Liu**[1], **Qing Li**[3], **Ying Guo**[2], **Lei Zhang**[2,4*], **Yan Yang**[1*]

[1] Institute of Edible Fungi, Shanghai Academy of Agricultural Sciences; National Engineering Research Center of Edible Fungi, Key Laboratory of Edible Fungi Resources and Utilization (South), Ministry of Agriculture, Shanghai 201403, China.

[2] Department of Pharmaceutical Botany, School of Pharmacy, Second Military Medical University, Shanghai, China.

[3] Department of Pharmacy, Changzheng Hospital, Second Military Medical University, Shanghai, 200003, China.

[4] State Key Laboratory of Subtropical Silviculture, Zhejiang A&F University, Hangzhou, Zhejiang 311300, China.

[#] These authors have contributed equally to this work.

[*]Correspondence:

Yan Yang, yangyan@saas.sh.cn; Tel./Fax: +86-21-6220-9765

Lei Zhang, zhanglei@smmu.edu.cn; Tel./Fax: +86-21-8187-1307

E-mail address:

Henan Zhang: henanhaoyun@126.com

Ruibing Chen: rbchenstar@163.com

Jingsong Zhang: 18918162047@189.cn

Qitao Bu: bujenny@163.com

Wenhan Wang: wangwenhan@saas.sh.cn

Yanfang Liu: aliu-1980@163.com

Qing Li: qli@smmu.edu.cn

Ying Guo: zoeytohar@hotmail.com

Lei Zhang: Leizhang100@163.com

Yan Yang: yangyan@saas.sh.cn

Extended Data Figure S1

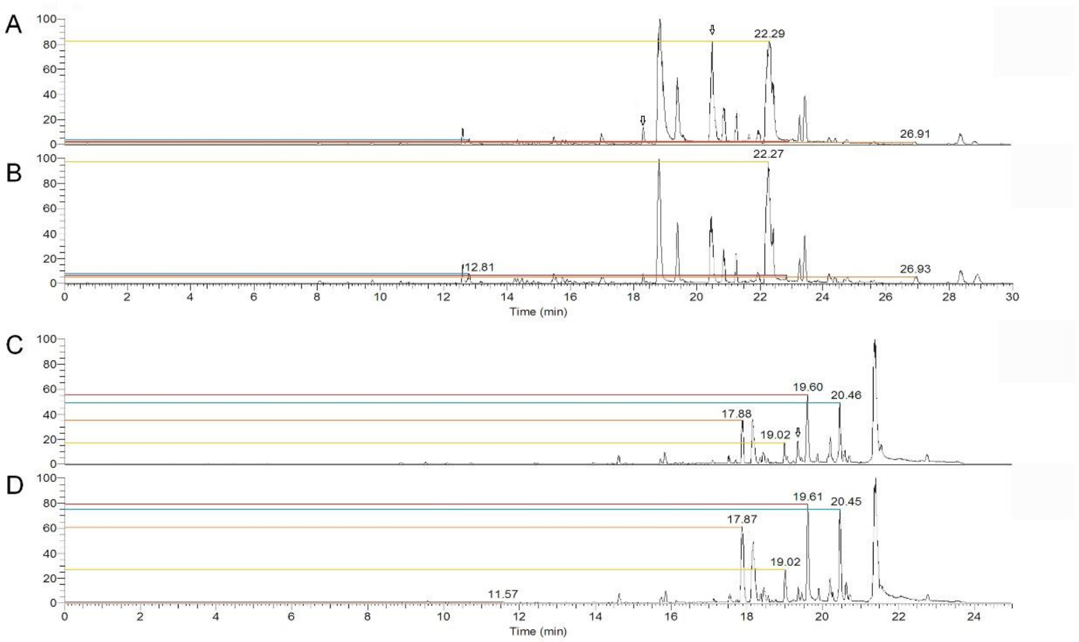

Extended Data Figure S2

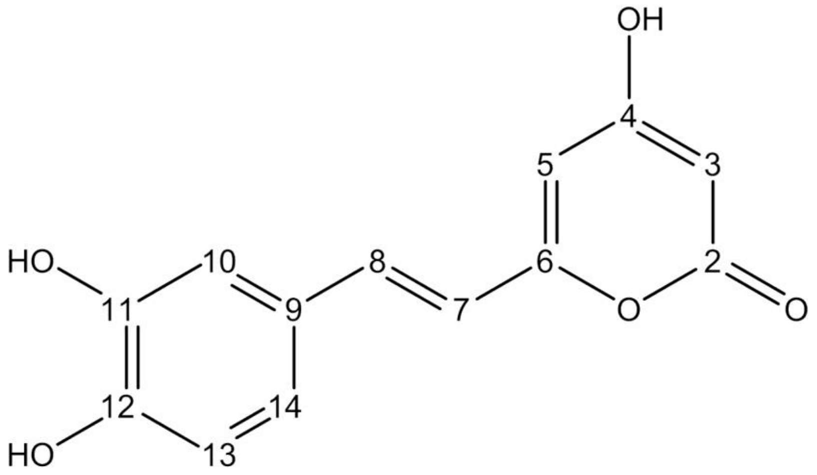

Extended Data Table S1

Type	A	N	R2X	R2Y	Q2
PCA-X	3	23	0.886	-	0.759
PLS-DA	2	20	0.651	0.964	0.913

Figure legends

Extended Data Figure S1. Typical base peak intensity chromatograms of urine samples. (A) SH1 strain, ESI+; (B) A67 strain, ESI+; (C) SH1 strain, ESI-; and (D) A67 strain, ESI-.

Extended Data Figure S2. NMR. Structure of hipidin purified from extracts as verified by nuclear magnetic resonance (NMR) analysis:^{1}H-NMR (300 MHz, DMSO-d6): δ 9.42 (OH); 9.04 (OH); 7.15 (d, 1, J = 16.1 Hz, H-8), 7.03 (d, 1, J = 1.9 Hz, H-10), 6.94 (dd, 1, J = 7.9, 1.9 Hz, H-14), 6.75 (d, 1, J = 8.1 Hz, H-13), 6.57 (d, 1, J = 16.1 Hz, H-7), 6.15 (d, 1, J = 1.9 Hz,H-5), 5.26 (d, 1, J = 1.9 Hz, H-3). ^{13}C NMR (75 MHz, DMSO-d6): 170.28 (C, C-2), 162.96 (CH, C-4), 159.73 (C,C-6), 147.34 (C, C-12), 145.54 (C, C-11), 134.58 (CH, C-8), 126.73 (C, C-9), 120.30 (CH, C-14), 116.31 (CH, C-13), 115.73 (CH, C-7), 114.06 (CH, C-10), 100.43 (CH, C-5), 89.20 (C, C-3).

Table legends

Extended Data Table S1. Parameters of PCA and PLS-DA models.

Extended Data Table S2. The list of differential metabolites in metabolome data. Red represents an increase in metabolite content and green represents a decrease in metabolite content (A67/SH1).

Extended Data Table S3. Fold changes of differential proteins between SH1 and A67. Red represents up-regulation and green represents down-regulation (A67/SH1).
